# A Comparison between Hi-C and 10X Genomics Linked Read Sequencing for Whole Genome Phasing in Hanwoo Cattle

**DOI:** 10.3390/genes11030332

**Published:** 2020-03-20

**Authors:** Krishnamoorthy Srikanth, Jong-Eun Park, Dajeong Lim, Jihye Cha, Sang-Rae Cho, In-Cheol Cho, Woncheoul Park

**Affiliations:** 1 Animal Genomics and Bioinformatics Division, National Institute of Animal Science, RDA, Wanju 55365, Korea; kris87@korea.kr (K.S.); jepark0105@korea.kr (J.-E.P.); lim.dj@korea.kr (D.L.); wischa91@korea.kr (J.C.); 2 Hanwoo Research Institute, National Institute of Animal Science, RDA, Pyeongchang 25340, Korea; chosr@korea.kr; 3 Subtropical Animal Research Institute, National Institute of Animal Science, RDA, Jeju 63242, Korea; choic4753@korea.kr

**Keywords:** Phasing, haplotypes, 10X genomics, Hi-C, Hanwoo, genome, SNPs

## Abstract

Until recently, genome-scale phasing was limited due to the short read sizes of sequence data. Though the use of long-read sequencing can overcome this limitation, they require extensive error correction. The emergence of technologies such as 10X genomics linked read sequencing and Hi-C which uses short-read sequencers along with library preparation protocols that facilitates long-read assemblies have greatly reduced the complexities of genome scale phasing. Moreover, it is possible to accurately assemble phased genome of individual samples using these methods. Therefore, in this study, we compared three phasing strategies which included two sample preparation methods along with the Long Ranger pipeline of 10X genomics and HapCut2 software, namely 10X-LG, 10X-HapCut2, and HiC-HapCut2 and assessed their performance and accuracy. We found that the 10X-LG had the best phasing performance amongst the method analyzed. They had the highest phasing rate (89.6%), longest adjusted N50 (1.24 Mb), and lowest switch error rate (0.07%). Moreover, the phasing accuracy and yield of the 10X-LG stayed over 90% for distances up to 4 Mb and 550 Kb respectively, which were considerably higher than 10X-HapCut2 and Hi-C Hapcut2. The results of this study will serve as a good reference for future benchmarking studies and also for reference-based imputation in Hanwoo.

## 1. Introduction

Advances in DNA sequencing technologies have made whole genome sequencing of individual cattle genomes possible [1]. Large-scale cattle genome sequencing (1000 bull genome project [2]) are helping us understand trait variant relationships. Haplotype phasing is the process of determining the sequences of genetic variants that cooccur along an intact maternal or paternal homologous chromosome [3,4]. Haplotype information is crucial for performing linkage analysis, association studies, population, and clinical genetic studies and also for allele specific impacts on gene expression [3,5]. Haplotype information are also critical for identifying heterozygous structural variants (SVs) [6]. A large number of strategies have been developed to generate phase information, including extensive laboratory-based protocols, de novo genome assembly strategies, and methods based on population haplotype frequency [7]. Most genome sequencing projects have used short-read sequencing. However studies in the human genome has shown that, although the data from small read sequencing has resulted in informative insights about small variants such as single nucleotide polymorphisms (SNPs), it has not captured the full spectrum of structural variations that exists [8]. Moreover, the loss of linkage information and mutations that are separated by distances longer than the read length generated from short-read sequencing severely limit their utility for phasing haplotypes [9].

These limitations can be overcome by long continuous reads technologies. “True” long-read sequencing technologies such as Pacific Biosciences Single Molecule Real-Time (SMRT) sequencing and nanopore technologies [10,11] have been immensely useful for applications such as genome assembly, but they require extensive computational error corrections [12,13]. As an alternative, technologies have emerged that leverage short-read sequencers which have low error rate along with sample preparation and processing for assembling long reads; these include Illumina TruSeq Synthetic Long-Reads [14], 10X genomics linked read sequencing [15], and chromosomal conformation and capture (proximity ligation and shotgun sequencing)-based Hi-C [16]. In linked read sequencing, barcoded libraries are generated, which are then sequenced using a short-read sequencer; the resulting reads are then assembled using the barcode information into long molecules called “Linked-Reads”. The same barcode information is then leveraged for phasing haplotypes, while Hi-C includes a set of experimental protocols that yields a sparse map of spatially proximally located read counts between pairs of loci [4]. Tools such as “HapCut” can leverage this distance information to recover long, high coverage accurate haplotypes [17,18]. The linked read sequencing and Hi-C technologies have been used for generating long phased haplotypes [19,20].

Hanwoo is the indigenous premium beef cattle of South Korea and has been extensively selected for higher meat and carcass quality traits. Efforts are underway for large-scale array-based genotyping for genomic selection and for predicting Genomic Estimated Breeding Values (GEBV). Haplotype-resolved whole genome sequence data will aid in imputing the array-generated SNP data to sequence level, which might help in capturing causal SNPs associated with traits of interest. Therefore, in this study, we performed haplotype phasing of a Hanwoo bull with data generated from the 10X genomics platform and Hi-C methods in three ways: (1) phasing of 10X genomics data using Long Ranger Pipeline, (2) phasing of 10X genomics data using HapCut2, and (3) phasing of Hi-C data using HapCut2. We then compared the phasing performance and accuracy of phasing using these approaches using a variety of metrics including switch error rates, pairwise single nucleotide variant (SNV) phasing accuracy and yield, and haplotype block length. The data generated in this study will be useful as a reference dataset for benchmarking future phased haplotypes from Hanwoo and in reference-based phasing and imputation with tools such as SHAPEIT [21].

## 2. Materials and Methods

**Figure 1:**
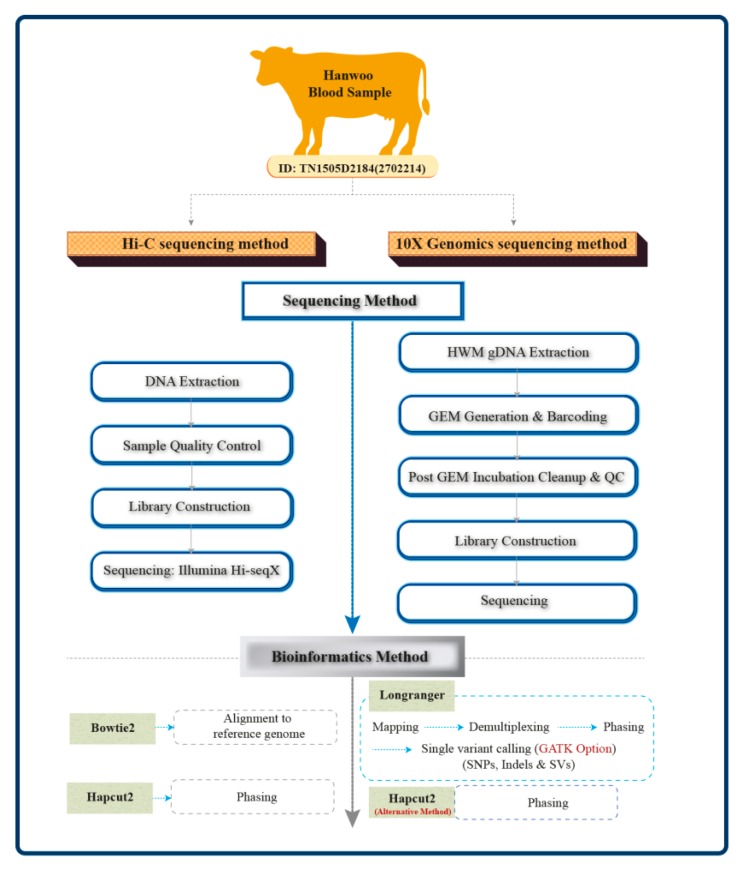
Schematic illustration of the two sample preparations and the three phasing approaches carried out in this study.

An overview of the methods followed in this study is given in Figure 1. 

### 2.1. DNA Sample

Blood sample was collected from a Proven Bull (KPN) (ID: TN1505D2184 (27214)). This bull was chosen as it had sired the largest number of offspring in the hanwoo breeding program. DNA was isolated from whole blood using the DNeasy Blood & Tissue Kit (Qiagen, Hilden, Germany) following the manufacturer’s guidelines. The size and quality of the isolated DNA was checked according to the manufacture’s recommendations [22]. 

### 2.2. 10X Genomics Sequencing and Analysis

First, the linked read library was prepared using the Chromium Genome Reagent Kit following the manufacturer’s protocol [23]. Briefly, 1.25 ng of the high molecular weight genomic DNA along with 10x Chromium reagents and gel breads were loaded onto a chromium controller chip. The input DNA was portioned into ~1 million droplets (GEMs), each containing a unique barcode (Gemcode). The droplets were then recovered from the chop and isothermally included at 30 °C for ~3 hrs to generate barcoded short reads; they were then purified and size-selected using Silane and Solid Phase reverse immobilization (SPRI) beads. Then, Illumina-compatible paired-end sequencing libraries were prepared following 10X Genomics recommendations. The libraries were quantified by qPCR (KAPA Biosystems Library Quantification Kit for Illumina platforms). Sequencing was conducted with an Illumina HiSeq X with 2 × 150 bp paired-end reads (Illumina, San Diego, CA, USA) following the manufacturer’s protocol. 

### 2.3. Hi-C Chromosome Conformation Captured Reads Sequencing

Approximately 100 μL of blood was cross-linked for Hi-C using previously described protocols [24]. The cross-linked cells were subsequently lysed, and nucleic acid was extracted as described previously [25]. The extracted DNA was digested with *Dpn*II (New England Biolabs, Ipswich, MA, USA) and cohesive end was repaired with biotinylated nucleotide with DNA polymerase I, followed by proximity ligation. The DNA was subsequently purified with QIAamp DNA mini Kit (Qiagen) following the manufacturers protocol. The purified DNA was sheared to a length of ~400 bp, and the biotinylated fragment was pulled down with Dynabeads MyOne Streptavidin C1 (Thermofisher, Waltham, MA, USA) following the manufacturers protocol. The Hi-C library for Illumina sequencing was prepared using NEBNext Ultra II DNA library Prep Kit for Illumina (NEB) according to the manufacturer’s instructions. The library was paired-end sequenced (2 × 150 bp) on Illumina HiSeq X platform. 

### 2.4. Read Assembly and Haplotype Phasing

All the runs were done on an Ubuntu (version 18.04.02) server with Intel^®^ Xenon^®^_CPU E5-2698 v4 @ 2.20 GHz (40 physical cores and 80 logical cores) and 1.5 TB of RAM.

The 10X sequencing data was analyzed using the Long Ranger pipeline [15] implemented by 10X genomics using default options. First, the reads were aligned to the bovine reference genome (UMD 3.1) using the Lariat aligner; subsequently, SNPs and insertion-deletion polymorphisms (indels) were called using the GATK mode (version 3.8) [26] within Long Ranger pipeline. The identified SNPs (Single nucleotide polymorphisms) were phased using two methods: first, using the phasing method implemented in Long Ranger, which builds on the Markov chain Monte Carlo (MCMC) algorithm-based phasing method proposed by Bansal et al. [27] by extending the probabilistic model to be robust to mixed fragments containing alleles from both haplotypes [28]. In the second method, the variants (SNPs) were phased using Hapcut2 [18] using default options. HapCut2 is an extension of the original Hapcut method [17] that performs haplotype assembly using DNA sequence fragments rather than population genotypes. HapCut2 uses a likelihood-based model which models and estimates platform specific errors. 

For the Hi-C data, we followed the GATK best practice pipeline to call a variant. First, the reads were aligned to the bovine reference genome (UMD 3.1) using BWA MEM (version 0.7.12) [29], the reads were then sorted, duplicates were marked, and the BAM file was indexed using Picard tools (version 2.9.2). SNPs and indels were called using HaplotypeCaller, implemented in GATK 3.8 [26]. Then, SNPs were phased using HapCut2 [18] using the default options. 

### 2.5. Metrics for Comparing Phasing Performance between the Platforms 

We had previously genotyped (with Illumina Bovine HD chip) and sequenced the individual used in this study (TN1505D2184 (27214)) using Illumina TruSeq synthetic long-read sequencing (Moleculo) and had generated a phased, error-corrected genome-scale haplotype. Briefly, based on the pedigree analysis of the Hanwoo population, we sequenced a trio (sire, dam, and offspring (TN1505D2184 (27214))) using Illumina Long-read haplotyping technology known as Moleculo; the generated short reads were assembled into synthetic long reads using BWA [29]. SNPs were called using default options in GATK [26]. The reads were phased into haplotypes using a two-step local and global phasing method [30]. Phasing accuracy was validated with array data (Illumina Bovine HD chip 777K) generated for the same animals. Results of this study are given in Table S1. We used this data as the gold standard for measuring phasing performance and accuracy of phasing of the data generated from the two platforms used in this study. We used four metrics for measuring phasing performance, including SER (Switch Error Rate), which measures inconsistences in phasing into correct haplotypes [7], and QAN50 (Quality Adjusted N50), which measures haplotype block length taking into account switch errors, thereby controlling for completeness and quality of phased haplotypes [31]. Phasing rate is the number of SNPs phased. Finally, we also measured pairwise SNV phasing accuracy and phasing yield, which measures the effect of distance between a pair of SNPs in a phasing block on phasing accuracy [3,32]. All the data generated in this study and the gold standards used for comparing are freely available for download from the National Agricultural Biotechnology Information Center (www.nabic.rda.go.kr) website under the following accession number: NV-0623. 

## 3. Results and Discussion

### 3.1. Sequencing and Variant Calling

A total of 790 million reads were generated using the 10X genomics platform at an average sequencing depth of 37.9X, out of which 95.7% (~756 million) of the reads were mapped to the reference genome, resulting in 8.8 million SNPs and 1.7 million indels (Table 1).

Out of the identified variants, 82% of the SNPs and 71% of the indels were heterozygous (Table 2). While 441 million reads at an average depth of 21.2X was generated with the Hi-C method, out of this, 438 million (99.2%) reads were mapped to the reference genome (UMD 3.1), resulting in 7.1 million SNPs and 1.7 million indels (Table 1), out of which 70% and 76% of the identified SNP and indels were heterozygous. The 10X and HiC approaches identified 89.4% and 71.32% of the SNPs in the gold standard (8,721,876 SNPs). 

### 3.2. Genome-Scale Haplotype Phasing

We explored three strategies for assembling haplotypes (Figure 1) and compared the results with the gold standard; 89.57% (7,766,580) of the SNPs identified by 10X genome sequencing (8,670,477 SNPs in total) were phased using the Long Ranger pipeline, which includes a phasing algorithm optimized for using 10X barcode information while phasing, while only 67.31% SNPs (5,836,541) were phased using HapCut2 (Table 3). Out of the 7,132,127 SNPs identified through Hi-C, only 51.65% (3,687,511 SNPs) was phased using HapCut2. The quality adjusted N50 (QAN50) length was longest for the 10X-LG at 1.2 Mb with a low SER (switch error rate) of 0.069%, followed by HiC-Hapcut2 (1.03Mb) and 10X-Hapcut2 (0.54 Mb); however, the HiC-Hapcut2 method had a higher SER of 0.24% than 10X-Hapcut2 (0.16%). The phasing accuracy achieved with all the three methods were comparable to the phasing accuracy reported previously in Holstein cattle using family-based and population-based methods [33]. 

### 3.3. Estimating Accuracy of Haplotype Phasing

**Figure 2:**
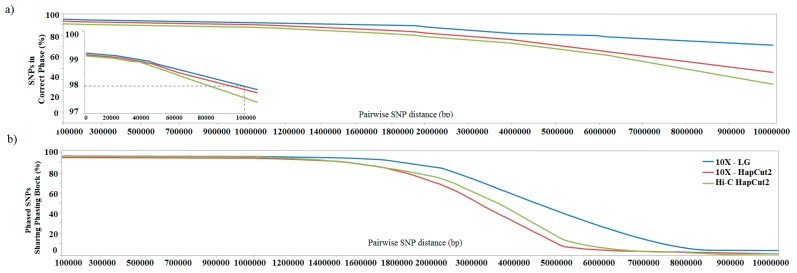
Comparison of phasing performance pairwise haplotype assignment: (**a**) Phasing accuracy shows the effect of distance on probability that SNPs on the same phasing block are correctly phased. (**b**) Phasing yield shows the effect of distance between a pair of SNPs on the probability that they are phased in the same phasing block.

We then measured the phasing accuracy and yield across pairs of SNPs (Figure 2) following the method reported by Snyder et al. [3]. Figure 2a shows pairwise SNP phasing accuracy of the three methods tested. The results showed that the phasing accuracy stayed above 98% (2 errors out of 100 heterozygous SNPs) for distances up to 72 Kb for HiC-Hapcut2, 93 Kb for 10X–HapCut2, and 100 Kb for 10X-LG. The phasing accuracy for 10X-LG stayed over 92% for distance up to 4 Mb. Figure 2b shows a phasing yield representing the probability that a pair of SNPs is phased in the same phasing block as a function of distance between the pair. Considering a distance up to 550 Kb, the phasing yield of 10X–HapCut2 and HiC-Hapcut2 remained over 90% and, for 10X-LG phasing, yielded over 90% extended up to 780 Kb. 

## 4. Conclusions

Our results show that linked-read data along with the Long Ranger pipeline has the best phasing performance, with high phasing rate, low switch error, and high phasing accuracy across distance. This is consistent with reports from human genome phasing, where linked-read data was shown to consistently outperform other methods [7]. Our analyses however have a few limitations such as the results of this study rely heavily on the accuracy of the gold standard used and only SNPs were considered in this study. Future studies must benchmark the ability of the sequencing platforms assessed in this study for identifying and phasing other variants such as short insertion-deletion polymorphisms (indels) and structural variations such as larger insertion, deletions, duplications, and copy number variants (CNVs) which may play important roles in many diseases. Moreover, haplotype-based genome-wide association studies are gaining popularity for identifying trait associated genes [34]. Therefore, the ability to phase diploid genomes using long, highly accurate sequence data along with low-cost computation will push haplotype-based genome analysis to higher levels within livestock genomics. Recent improvements in Pacific Biosciences SMRT sequencing and Oxford nanopore sequencing are suggested to have improved haplotype phasing quality [35,36]; therefore, future studies could evaluate the performance of these technologies for accurately assemble genome scale haplotypes.

## Figures and Tables

**Table 1:** Summary of sequencing data from the two platforms.

	10X Genomics	Hi-C
Total Reads	790,643,590	441,889,616
Mapped Reads	756,645,916 (95.7%)	438,995,090 (99.2%)
Q30 (%)	92.50%	93.00%
Mean Depth	37.9X	21.2X

**Table 2:** Summary of single nucleotide polymorphisms (SNPs) and insertion-deletion polymorphisms (indels) identified in this study.

	10X Genomics		Hi-C	
	SNPs	Indels	SNPs	Indels
**Total**	8,670,477	1,749,472	7,132,127	1,753,086
**Homozygous (%)**	2,590,042 (30%)	507,761 (29%)	2,170,128 (30%)	417,903 (24%)
**Heterozygous (%)**	6,080,435 (70%)	1,241,711 (71%)	4,397,837 (70%)	1,335,183 (76%)

**Table 3:** Summary of phasing performance: Metrics shown are total number of SNPs phased, percentage of SNPs phased, Quality adjusted N50 (QAN50), and the switch error rate.

Sequencing Platform—Phasing Method	Metrics for Phasing Performance	
10X-LG	Total SNPs Phased	7,766,580
	% of SNPs Phased	89.57
	QAN50 (bp)	1,249,365
	SER * (%)	0.07
10X–HapCut2	Total SNPs Phased	5,836,541
	% of SNPs Phased	67.31
	QAN50 (bp)	541,912
	SER * (%)	0.16
Hi-C–HapCut2	Total SNPs Phased	3,687,511
	% of SNPs Phased	51.65
	QAN50 (bp)	1,034,586
	SER *(%)	0.24

* Switch Error Rate.

## Supplementary Materials

The following are available online at https://www.mdpi.com/2073-4425/11/3/332/s1, Table S1: Summary of variants identified and phased in the reference library through Illumina Synthetic Long Read Sequencing.
