# Supplementary material for: A Comparison between Hi-C and 10X Genomics Linked Read Sequencing for Whole Genome Phasing in Hanwoo Cattle
Source: Genes (Basel). 2020 Mar 20;11(3):332. doi: 10.3390/genes11030332 (PMC7140831; doi:10.3390/genes11030332)
Supplement: Supplementary file 1 [file genes-11-00332-s001.pdf]

Table S1. Summary of variants identified and phased in the reference library through Illumina Synthetic long read Sequencing.

Sample Name	SNP			INDEL			Total(%)
	Het	Phased Het	(%)	Het	Phased Het	(%)	
**TN1503D1092(29058)**	3,314,156	2,898,494	87.46%	398,626	357,830	89.77%	87.71%
**TN1503D1093(12007)**	4,000,577	3,161,407	79.02%	446,110	370,534	83.06%	79.43%
**TN1505D2184(27214)**	3,842,027	2,666,109	69.39%	429,455	322,914	75.19%	69.98%
